# Use of nutritional supplements by elite Japanese track and field athletes

**DOI:** 10.1186/s12970-020-00370-9

**Published:** 2020-07-22

**Authors:** Shogo Tabata, Fumihiro Yamasawa, Suguru Torii, Tomohiro Manabe, Hiroshi Kamada, Akira Namba, Jo Kato, Haruka Kaneko, Keitaro Tahara, Yuka Tsukahara, Kazuki Sato

**Affiliations:** 1 Institute for Integrated Sports Medicine, Keio University School of Medicine, Shinanomachi 35, Shinjuku, Tokyo, Japan; 2 Medical Committee, Japan Association of Athletics Federations (JAAF), Kasumigaokamachi 4-2 Japan Sport Olympic Square 9th floor, Shinjuku, Tokyo, Japan; 3 Faculty of Sport Sciences, Waseda University, Mikajima 2-579-15, Tokorozawa, Saitama, Japan; 4 Sports Medicine Research Center, Keio University, Hiyoshi 4-1-1, Kohoku, Yokohama, Kanagawa Japan; 5 Department of Orthopaedic Surgery, University of Tsukuba, Amakubo 2-1-1, Tsukuba, Ibaraki, Japan; 6 Department of Clinical Genomics, Saitama Medical University, Morohongo 38, Moroyama, Iruma, Saitama, Japan; 7 Department of Cardiology, National Hospital Organization Kasumigaura Medical Center, Shimotakatsu 2-17-14, Tsuchiura, Ibaraki, Japan; 8 Department of Medicine for Orthopedics and Motor Organ, Juntendo University Graduate School of Medicine, Hongo 3-1-3, Bunkyo, Tokyo, Japan; 9 Department of Orthopaedics Surgery, Tokyo Metropolitan Tama Medical Center, Musashinodai 2-8-29, Fuchu, Tokyo, Japan

**Keywords:** Track and field, Supplements, Japanese athletes, Amino acids, Vitamins, Minerals, Ergogenic aids, ADRV

## Abstract

**Background:**

While scientific evidence supports the efficacy of only limited nutritional supplements (NS) on sports performance, the use of NS is widespread in athletes. Given the serious issues of health damage or unintended Anti-Doping Rule Violations due to ingestion of contaminated NS in sports, accurately understanding NS practices by athletes is crucial. This study therefore elucidated the use of NS by elite Japanese track and field (TF) athletes.

**Methods:**

The subjects were 574 Japanese TF athletes, including 275 junior athletes (under 20 years) and 299 senior athletes, who participated in international competitions from 2013 to 2018. Data on NS use were collected through pre-participation medical forms obtained from all entrants before their participation in competitions. NS users were requested to report the product names and primary components of all NS they were taking.

**Results:**

The overall prevalence of NS use was 63.9%. The mean number of NS products used per athlete was 1.4. The prevalence was significantly higher in women (69.2%) than in men (59.6%) (*p* = 0.018) and significantly higher in senior athletes (68.9%) than in junior athletes (58.9%) (*p* = 0.012). The prevalence of NS use was higher in long-distance runners (75.8%) and lower in jumpers (52.3%) and throwers (49.2%) than other disciplines (*p* < 0.001). The most prevalent components were amino acids (49.3%), followed by vitamins (48.3%), minerals (22.8%), and protein (17.8%).

**Conclusions:**

Approximately two-thirds of elite Japanese TF athletes reported the use of NS, and NS practices varied by gender, age, and discipline.

## Background

The use of nutritional supplements (NS) has become widespread among athletes at all levels, despite their use not being considered necessary for athletes who consume an adequate diet (excluding those on restricted energy intake for weight control) [1]. As NS have been defined and categorized in many ways, no standardized definitions or classifications have yet been established. For example, the Dietary Supplement Health and Education Act of 1994 (DSHEA) defines a dietary supplement as, “a product (other than tobacco) that is intended to supplement the diet that contains one or more of the following dietary ingredients: a vitamin, a mineral, an herb or other botanical, an amino acid, a dietary substance for use by man to supplement the diet by increasing the total dietary intake, or a concentrate, metabolite, constituent, extract or combination of any of the ingredients listed above” [2]. However, in the International Olympic Committee (IOC)‘s 2018 consensus statement, the definition of DSHEA was considered unsatisfactory, as this description depended on whether or not a ‘healthy diet’ had been consumed; the IOC therefore defined a dietary supplement as “a food, food component, nutrient, or non-food compound that is purposefully ingested in addition to the habitually consumed diet with the aim of achieving a specific health and/or performance benefit” [3].

NS can generally be classified as 1) dietary supplements (e.g. vitamins or minerals) and sports foods (e.g., sports drinks, energy bars, or proteins) that can be used to gain health benefits or to correct or prevent nutrient and/or energy deficiencies due to an inadequate diet; and 2) ergogenic aids (e.g. caffeine, creatine monohydrate, or amino acids) that can be used to enhance sports performance.

Although the absence of globally standardized definitions of NS hampers accurate determination of the prevalence of NS use in sports, the use of NS within athletic populations is nevertheless reported to be relatively high [4]. Indeed, a recent systematic review and meta-analysis targeting athletes reported an overall prevalence of NS use of approximately 60% among various sporting groups [5]. However, the prevalence and types of NS being used vary substantially depending on the type of sport, level of competition, athlete gender and age, and region [1, 6–11]. For example, a recent study targeting elite Spanish athletes reported that body building, cycling, track and field (TF), triathlon, and aquatics were sports disciplines with high proportions of athletes using NS [8]. Based on their systematic review and meta-analysis, Knapik et al. reported that elite athletes used NS much more frequently than their non-elite counterparts [5].

Several studies have reported that vitamin or mineral supplements have no marked positive effects on sports performance in athletes without deficiencies in those nutritional elements [12–14]. Further, the International Society of Sports Nutrition (ISSN) exercise & sports nutrition review states that only a few ergogenic aids have strong evidence supporting their safety and efficacy for muscle building and performance enhancement [15]. The IOC consensus statement presented a similar perspective, stating that only a few NS have strong evidence supporting their utility in benefiting athletes’ performance when used in certain scenarios [3].

In addition to the likelihood of limited benefit, the risk of health damage due to the ingestion of NS contaminated with unapproved ingredients must also be considered. For example, the US Food and Drug Administration (FDA) found that 776 dietary supplements marketed for sexual enhancement, weight loss, or muscle building had been adulterated with unapproved pharmaceutical ingredients, and implicated 146 different dietary supplement companies in this adulteration from 2007 to 2016 [16]. In addition, the number of cases of unintended Anti-Doping Rule Violations (ADRVs) associated with the use of NS containing unlabeled substances banned by anti-doping regulations and organizations have been increasing. An international survey by the IOC revealed that, of 634 NS samples analyzed, 94 (14.8%) contained anabolic androgenic steroids not declared on the label [17]. Athletes and coaches must be aware of the issues associated with the use of any contaminated NS, and should select NS carefully with a full understanding of the contents and confirmation of the claims made by the product [18] in order to safeguard athletes’ health and prevent unintended ADRVs. However, previous studies have reported that most athletes are unaware of issues of contamination or side effects associated with NS [19–21]. A review reported that the proportion of ADRVs that might be attributed to contaminated NS use ranged from 6.4 to 8.8% [22]. Japanese athletes are reported to have a higher proportion of ADRVs due to NS use (about 30%) than athletes in Western countries, based on reports published on the Japan Anti-Doping Agency (JADA)‘s official website [23]. According to the 2016 ADRV report published by the World Anti-Doping Agency (WADA), TF athletes showed the highest number of ADRVs [24]. As mentioned above, the high prevalence of NS use in TF athletes has also been reported [8]; however, few studies have investigated the use of NS in TF athletes [1, 6]. Although a previous study found that Asian TF athletes consumed fewer NS than athletes from other continents [1], to our knowledge, the prevalence of NS use among elite Japanese athletes, particularly TF athletes, has not been fully investigated. We also hypothesized that assessing the various disciplines included in TF might be useful for clarifying the differences in NS use by athletic characteristics.

Therefore, the present study evaluated the use of NS among elite Japanese TF athletes.

## Methods

### Study population

The subjects in the present study were 275 junior (< 20 years old) (mean age ± standard deviation [SD]: 17.7 ± 1.1 years) and 299 senior (25.2 ± 3.9 years) Japanese TF athletes who, as members of Japanese national teams, competed at 38 international TF competitions from July 2013 to October 2018. The athletes completed a pre-participation medical form (PMF). Athletes who participated in multiple teams were counted only once, and the most recent PMF was used.

Disciplines were divided into 7 categories as follows: sprinting (≤400 m), middle-distance (800–1500 m), long-distance (≥3000 m, 3000 m steeplechase, marathon, and race walking), hurdles, jumping (long jump, triple jump, high jump, and pole vault), throwing (shot put, discus throw, hammer throw, and javelin throw), and combined events (heptathlon and decathlon). Table 1 shows the number of subjects included among discipline categories.

**Table 1:** Number of subjects by discipline

Discipline	Junior (*n* = 275)		Senior (*n* = 299)		Total (*n* = 574)	
	Men	Women	Men	Women	Men	Women
Sprinting	30	23	30	14	60	37
Middle-distance	9	9	14	14	23	23
Long-distance	49	42	64	64	113	206
Hurdle	15	10	16	6	31	16
Jumping	28	20	22	16	50	36
Throwing	17	18	13	17	30	35
Combined events	3	2	4	5	7	7
Total	151	124	163	136	314	260

### Pre-participation medical form

The paper-based PMF was sent to all entrants by the Japan Association of Athletics Federations (JAAF) Medical Committee before their participation in any international competition. On the PMF, athletes were asked, “Are you taking any NS?”. Athletes who answered “Yes” were asked to report all product names and the primary components of the NS they were using. The term “NS” was not explicitly defined, as the definition was considered to vary among individual respondents. On completing the form, athletes were required to fax or mail the PMF to the JAAF office.

We extracted the data related to NS use from the PMF and conducted a retrospective evaluation. The option to opt out was provided via the official JAAF website (https://www.jaaf.or.jp), which also provided easily accessible information on this study and the right to refuse to participate. All protocols were approved by the Research Ethics Committee of Keio University in Tokyo, Japan (#20180294).

### Nutritional supplement classifications

NS were divided into 11 categories by referencing the supplement classification of the Japan Institute of Sports Sciences (JISS) [11], as follows: protein, carbohydrate, vitamins, minerals, amino acids, creatine, caffeine, fish oil, ubiquinone, herbal supplements, and others. The main components of each product were checked using the product information webpage of the relevant manufacturing company to ensure no mistakes had been made. NS containing multiple components were placed in multiple categories. For instance, a product containing both multi-vitamins and multi-minerals as the main components was placed into both the vitamins and minerals categories.

### Statistical analyses

The data were analyzed using frequency distributions and cross-tabulations. The chi-squared test was used to assess differences in the prevalence of NS use by gender, age, and discipline. When tests showed significance, differences were specified using an adjusted residual analysis, with an adjusted residual of 1.96 regarded as significant at the 5% level. Data that showed expected frequencies of ≤5 were excluded. The Mann-Whitney U test was used to analyze differences in the number of NS products used per athlete by gender and age. The Kruskal-Wallis test was used to analyze differences in the number of NS products used among disciplines.

Data were reported as the mean ± standard deviation (SD). Statistical analyses were performed using IBM SPSS Statistics for Mac OS (Ver. 25; IBM Japan, Tokyo, Japan). A *p*-value of less than 0.05 was considered statistically significant.

## Results

The overall prevalence of NS use in athletes was 63.9%. The chi-squared test showed that prevalence was significantly higher in women (69.2%) than in men (59.6%), as well significantly higher in senior athletes (68.9%) than in junior athletes (58.9%). By discipline, the adjusted residual analysis revealed that the prevalence of NS use was significantly higher in long-distance runners (75.8%) and lower in jumpers (52.3%) and throwers (49.2%) than in other disciplines (Table 2).

**Table 2:** Prevalence of each component use by gender, age, and discipline

	Any NS	Protein	Carbohydrate	Vitamins	Minerals	Amino acids	Creatine	Caffeine	Fish oil	Ubiquinone	Herbal	Others
**Total**	63.9% (367)	17.8% (102)	5.9% (34)	48.3% (277)	22.8% (131)	49.3% (283)	9.2% (53)	1.2% (7)	5.1% (29)	3.5% (20)	5.7% (33)	16.2% (93)
**Gender**												
Male	59.6% (187)*	20.7% (65)*	4.1% (13)	43.3% (133)*	19.7% (62)	43.3% (136)*	12.1% (38)*	1.6% (5)	4.1% (13)	2.2% (7)	4.8% (15)	13.7% (43)
Female	69.2% (180)	14.2% (37)	8.1% (21)	55.4% (144)	26.5% (69)	56.5% (147)	5.8% (15)	0.8% (2)	6.2% (16)	5.0% (13)	6.9% (18)	19.2% (50)
*p-value*	*0.018*	*0.048*	*n.s.*	*0.002*	*n.s.*	*0.002*	*0.009*	*n.s.*	*n.s.*	*n.s.*	*n.s.*	*n.s.*
**Ages**												
Junior	58.9% (161)†	14.9% (41)	6.2% (17)	42.2% (116)†	16.4% (45)†	44.7% (123)†	5.8% (16)†	0.7% (2)	1.5% (4)†	2.5% (7)	4.0% (11)	11.6% (32)†
Senior	68.9% (206)	20.4% (61)	5.7% (17)	53.8% (161)	28.8% (86)	53.5% (160)	12.4% (37)	1.7% (5)	8.4% (25)	4.3% (13)	7.4% (22)	20.4% (61)
*p-value*	*0.012*	*n.s.*	*n.s.*	*0.006*	*< 0.001*	*0.037*	*0.009*	*n.s.*	*< 0.001*	*n.s.*	*n.s.*	*n.s.*
**Discipline**												
Sprinting	57.7% (56)	16.4% (16)	3.1% (3)	38.1% (37)§	10.3% (10)§	42.3% (41)	21.6% (21)‡	2.1% (2)	0% (0)§	0% (0)§	2.1% (2)	11.3% (11)
Middle-distance	71.7% (33)	10.9% (5)	6.5% (3)	54.3% (25)	21.7% (10)	58.7% (27)	0% (0)§	4.3% (2)	4.3% (2)	4.3% (2)	6.5% (3)	15.2% (7)
Long-distance	75.8% (166)‡	15.5% (34)	6.8% (15)	60.3% (132)‡	38.4% (84)‡	58.9% (129)‡	0.9% (2)§	0.5% (1)	11.0% (24)‡	7.3% (15)‡	11.4% (25)‡	25.1% (55)‡
Hurdle	57.4% (27)	17.0% (8)	8.5% (4)	44.7% (21)	10.6% (5)§	38.3% (18)	12.8% (6)	2.0% (1)	6.4% (3)	2.1% (1)	2.1% (1)	12.8% (6)
Jumping	52.3% (45)§	16.3% (14)	5.8% (5)	47.7% (41)	18.6% (16)	43.0% (37)	5.8% (5)	1.2% (1)	0% (0)§	1.2% (1)	1.2% (1)§	10.5% (9)
Throwing	49.2% (32)§	35.4% (23)‡	6.1% (4)	21.5% (14)§	7.7% (5)§	36.9% (24)§	26.2% (17)‡	0% (0)	0% (0)§	1.5% (1)	0% (0)§	4.6% (3)§
Combined events	44.4% (8)	14.3% (2)	0% (0)	50.0% (7)	7.1% (1)	50.0% (7)	14.3% (2)	0% (0)	0% (0)	0% (0)	7.1% (1)	14.3% (2)
*p-value*	*< 0.001*	*0.012*	*n.s.*	*< 0.001*	*< 0.001*	*0.004*	*< 0.001*	*n.s.*	*< 0.001*	*0.031*	*0.001*	*0.001*

*NS* Nutritional supplement; *n.s.* Not significant

The chi-square test was used to assess differences in the prevalence of each component use by gender, age, and discipline

The chi-square test *p*-value is shown. *p < 0.05 vs. women, †*p* < 0.05 vs. seniors

The specific significance determined by an adjusted residual analysis was expressed as ‡ (higher) or § (lower)

The chi-square test *p*-value is shown

A total of 817 products were used, with a mean 1.4 NS products used per athlete. The maximum number of products used (12 products) was reported in a female senior marathon runner. According to the Mann-Whitney U-test, the mean number of NS products used per athlete was higher in women than in men (1.6 vs. 1.3, *p* = 0.008) and in senior than in junior athletes (1.8 vs. 1.0, *p* < 0.001) significantly. According to the Kruskal-Wallis test between disciplines, long-distance runners reported using more NS products per athlete than sprinters, jumpers, and throwers (1.9 for long-distance vs. 1.1, 1.1, and 1.1 for sprinting, jumping, and throwing; *p* = 0.003, 0.001, and 0.008, respectively) (Table 3).

**Table 3:** Number of NS products used per athlete

	mean ± SD
**Total**	1.4 ± 1.6
**Gender**	
Males	1.3 ± 1.4
Females	1.6 ± 1.7
*p-value*	*0.008*
**Age groups**	
Junior	1.0 ± 1.2
Senior	1.8 ± 1.8
*p-value*	*< 0.001*
**Disciplines**	
Sprinting	1.1 ± 1.2*
Middle-distance	1.3 ± 1.3
Long-distance	1.9 ± 1.8
Hurdle	1.3 ± 1.4
Jumping	1.1 ± 1.5*
Throwing	1.1 ± 1.3*
Combined events	1.3 ± 1.5

*SD* Standard deviation

The Mann-Whitney U test was used to analyze differences by gender and age. The *p*-values are shown

The Kruskal-Wallis test was used to analyze differences among disciplines. *p < 0.05 vs. long-distance

Table 2 shows the prevalence of use of each component by gender, age, and discipline. The most prevalent components were amino acids (49.8%), followed by vitamins (48.3%), minerals (22.8%), and proteins (17.8%). Table 4 shows a breakdown of “other” components. Men showed a significantly higher prevalence of protein (20.7%) and creatine (12.1%) as components than women (14.2 and 5.5%, respectively), who conversely showed a significantly higher prevalence of vitamins (55.4%) and amino acids (56.5%) than men (43.3 and 43.3%, respectively). Senior athletes showed a significantly higher prevalence of vitamins (53.8%), minerals (28.8%), amino acids (53.5%), creatine (12.4%), fish oil (8.4%), and “other” components (20.4%) than junior athletes (42.2, 16.4, 44.7, 5.8, 1.5, and 11.6%, respectively). Long-distance runners showed a significantly higher prevalence of vitamins (60.3%), minerals (38.4%), amino acids (58.9%), ubiquinone (7.3%), fish oil (11.0%), herbal supplements (11.4%), and “other” components (25.1%) than other disciplines but a significantly lower prevalence of creatine (0.9%). Throwers, by contrast, showed a significantly higher prevalence of protein (35.9%) and creatine (26.2%) but a significantly lower prevalence of vitamins (21.5%), minerals (7.7%), amino acids (36.9%), fish oil (0%), herbal supplements (0%), and “other” components (4.6%) than other disciplines. Sprinters showed a higher prevalence of creatine (21.6%) but a significantly lower prevalence of vitamins (38.1%), minerals (10.3%), ubiquinone (0%), and fish oil (0%) than other disciplines. Hurdlers showed a significantly lower prevalence of minerals (10.6%), middle-distance runners showed a significantly lower prevalence of creatine (0%), and jumpers showed a significantly lower prevalence of fish oil (0%) and herbal supplements (1.2%) than other disciplines.

**Table 4:** Main components categorized as “Others”

	number
Citric acid	21
Casein phosphopeptide (CPP)	18
Lactic acid bacterium	17
Taurine	10
Enzyme	7
Bee extract	7
Astaxanthin	6
Glucosamine	6
Collagen	5
Chondroitin	4
Yeast	4
Liver extract	4
Beta-carotene	3
Royal jelly	3
Alpha lipoic acid	2
Melatonin	1
Elastin	1
Propolis	1
Phosphatidylserine	1
Transfer factor	1
alpha-Glycerylphosphorylcholine (α-GPC)	1
Methylsulfonylmethane (MSM)	1

## Discussion

To our knowledge, this study is the first to investigate the prevalence of NS use and differences in NS use by gender, age, and discipline among elite Japanese TF athletes. As the principal result, we found that 63.9% of elite Japanese TF athletes took some kind of NS. While only a few studies have reported the prevalence of NS use in TF athletes, the present result is consistent with the previous finding that the prevalence of NS use in top-level TF athletes was 66.8%, according to an analysis of doping control forms collected during 12 World Athletics (WA) World Championships and 1 out-of-competition season [1]. Tscholl et al. reported that the prevalence of NS use among elite football players in the Fédération Internationale de Football Association (FIFA) World Cup was 57.1%, which was lower than that found in TF athletes [7]. Several studies have also found that TF athletes tended to report a high prevalence of NS use compared with athletes involved in other sports [8, 11]. Physique and morphological characteristics play important roles in competition success among TF athletes [25]. The relatively high prevalence of NS use among TF athletes might therefore be reasonable.

While Asian TF athletes were reported to consume fewer NS (mean number of products per athletes: 1.1) than athletes from other continents, except for Africa (1.9, 1.9, 2.2, and 2.0 for Europe, North America, Oceania, and South America, respectively), that study included many developing countries in Asia, and a country-by-country analysis was not conducted [1]. The mean number of NS products used per athlete in the present study was 1.4. The JISS reported that the mean number of NS products used per TF athlete who participated in the 2012 London Olympic Games was 1.7 [11]. These findings clearly show that Japanese elite athletes tend to use NS much more frequently than those of other Asian countries.

The present study also revealed that the prevalence of NS use differed by gender and age. More women (69.2%) reported the use of NS than men (59.6%), as was also noted in the previous report from the IAAF [1]. Further, gender differences were also seen in the prevalence of components. Women showed a higher prevalence than men of using vitamins or minerals, while men showed a higher prevalence than women of using protein, creatine, and caffeine.

According to the analysis by age category, senior athletes showed a higher prevalence of all NS components (except for carbohydrates) (68.9%) than junior athletes (58.9%), indicating that the prevalence of NS use increased with increasing competitive level. This finding was further supported by the higher prevalence of NS use (86.2%) among Japanese TF Olympic athletes at the highest competition level [11] than among athletes in the present study. However, the prevalence of NS use among Japanese junior TF athletes (58.9%) was nearly 3 times that among general Japanese high school students [26]. Neiper et al. also reported that the prevalence of NS use was 62% among elite junior TF athletes in the UK, with a trend toward more frequent use by women (75%) than men (55%) [6]. These findings suggest that NS use is widespread among not only senior athletes but junior athletes as well, although safety data concerning the use of NS by young populations are largely lacking [27, 28].

While a previous study showed a lower prevalence of NS use among middle- and long-distance athletes than those who participate in power events, such as sprinting, throwing, and jumping [1], we obtained the opposite result in the present study, with the prevalence of NS use being significantly higher among long-distance athletes than those who participated in other disciplines. Indeed, three-quarters of long-distance runners evaluated in the present study were taking some kind of NS. In particular, the rate of mineral use was two to five times higher among long-distance runners than those participating in other disciplines. The high prevalence of iron deficiency anemia in Japanese endurance athletes might be contributing to the frequent use of NS containing minerals, including iron, by long-distance runners. The JAAF medical committee surprisingly reported that the self-assessed incidence rate of anemia among middle- and long-distance runners at elite Japanese high schools was relatively high, at 31.5% in boys and 46.1% in girls [29]. Tscholl et al. similarly reported the frequent use of iron supplements among middle- and long-distance runners [1].

Almost half of the elite Japanese TF athletes in this study reported the use of amino acids and vitamins, which were the most prevalent components in the present study. This result was attributed to the frequent use of a specific product containing branched-chain amino acids (BCAAs; leucine, isoleucine, and valine) and multivitamins that is popular among Japanese athletes and has been actively promoted to them. The JISS also reported that amino acids were the most commonly used agent among Japanese elite athletes [11, 30]. However, only limited evidence exists to support the hypothesis that BCAA supplementation during intense training helps minimize protein degradation and thus leads to greater gains in fat-free mass [15, 31–33]. In the ISSN exercise & sports nutrition review, essential amino acids (EAAs) are categorized as supplements with strong evidence supporting their efficacy and are apparently safe only for muscle building; however, BCAAs are categorized as supplements with limited or mixed evidence supporting their efficacy for muscle building and performance enhancement [15]. Of note, the effect of BCAA supplementation in stimulating an increase in muscle protein synthesis was notably weaker than that of full component EAA [15]. Moreover, several studies have reported that BCAA supplementation was ineffective for improving performance and reducing skeletal muscle damage in endurance events [34, 35]. Considering the limited evidence supporting the efficacy of amino acids in endurance events, the finding that Japanese elite long-distance runners tend to consume NS containing amino acids warrants further investigation.

Although vitamin and mineral supplementation is not necessary for athletes with an adequate diet [12–14], such supplementation might be warranted for female athletes at risk of vitamin or mineral insufficiency due to inadequate dietary intake, menstruation, or inflammatory responses to heavy physical activity [36]. Indeed, the women in the present study tended to show a higher prevalence of vitamin and mineral consumption than men. However, further studies should clarify whether such supplementations are necessary with consideration to dietary intake, nutritional status, and exercise volume.

Among the ergogenic aids included in NS in the present study, creatine and caffeine have been classified as supplements with strong evidence supporting their efficacy in enhancing sports performance [3, 15]. Studies have consistently shown that creatine monohydrate supplementation increases intramuscular creatine concentrations, which may help explain the observed improvements in high-intensity exercise performance, thus leading to greater training performance [37]. The most effective way to increase muscle creatine stores is to ingest 5 g of creatine monohydrate (or approximately 0.3 g/kg body mass) 4 times daily for 5–7 days, followed by 3–5 g/day thereafter in order to maintain elevated creatine levels [3, 15, 37]. Creatine supplementation with long-term use appears to have no negative health effects in healthy individuals when appropriate loading protocols are followed [38, 39]; however, a potential small increase in body mass after creatine loading as a result of water retention may be detrimental for sports with weight classes/restrictions or where an increased body mass may decrease performance, such as in long-distance and jumping events [3, 40]. The WA Consensus Statement 2019 (Nutrition for Athletics) noted that creatine supplementation may help achieve a marginal performance gain in all TF disciplines except for middle- (1500 m) and long-distance events [41, 42]. This notion is consistent with the results of the present study, where approximately one-quarter of sprinters and throwers but no middle-distance runners and only a few long-distance runners reported the use of creatine.

Caffeine has also been shown to be an effective ergogenic aid that possesses well-established benefits for athletic performance across endurance-based situations and short-term, supramaximal, and/or repeated sprint tasks when 3–6 mg/kg of body mass is consumed within 60 min before and/or during exercise in the form of anhydrous caffeine (i.e. pill or powder form) [3, 15, 43–46]. The IAAF also regards caffeine as an effective ergogenic aid for performance enhancement in all TF disciplines [41, 42]. A review and meta-analysis showed that the consumption of caffeine-containing energy drinks improved performance in several physical and sport situations that included muscle strength protocols, jumping, endurance exercise tests, and sports-specific actions [47]. Another review reported that caffeinated coffee may also be used as a safe alternative to anhydrous caffeine to improve endurance performance [44]. Although the prevalence of caffeine-containing NS use was lowest, at only 1.2% in the present study, despite strong evidence of positive impact on sports performance, prevalence might have been higher if consuming caffeine-containing beverages, such as energy drinks, coffee, or tea, had been counted as caffeine supplementation. However, increased caffeine doses should be avoided, as caffeine doses over ≥9 mg/kg body mass do not appear to increase the performance benefit and are instead likely to increase the risk of negative side effects, including nausea, anxiety, insomnia, and restlessness [3, 48, 49].

The WA also listed nitrate/beetroot juice, beta-alanine, and sodium bicarbonate as well as creatine and caffeine as performance supplements that may help improve performance in TF events [41, 42]; however, no athletes in the present study reported the use of such supplements, given that nitrate, beta-alanine, and sodium bicarbonate are relatively unfamiliar to Japanese populations. Considering the gap between the prevalence of use of a given NS and the evidence supporting its efficacy, athletes may very well consume NS without closely evaluating their efficacy on improving performance in events or safety.

While we did not evaluate the reasons for the use of NS, Garthe and Maughan et al. reported that some athletes consumed NS not only for performance and/or health benefits but also for other reasons, such as a “just in case” insurance policy, financial gain (sponsorship), or on advice from other parties, including coaches, fellow athletes, parents, and marketing [4]. A marked influence of coaches, managers, and trainers on supplementation practices was also reported in a previous study targeting Japanese Olympians [11]. In addition, a lack of knowledge concerning active ingredients, mechanisms of action, recommended doses, and adverse effects of NS has been reported among athletes [20, 50]. Surprisingly, Braun et al. found that only 36% of elite young German athletes were aware of the issue of supplement contamination [19]. To prevent adverse effects or unintended ARDVs due to using contaminated NS, athletes must be alerted to these risks and advised to take precautions against the blind consumption of NS on the advice of individuals other than themselves, or the unnecessary use of NS without evidence.

One limitation associated with the present study was our method of evaluating NS use. The prevalence of NS use was assessed based on response to the PMF by the athletes themselves, and no objective methods were practiced. Further, differences among individuals in the definitions of NS might have led to underestimation of the prevalence of NS use. Variations in the number of subjects by discipline may also have been a limitation. Finally, since we assessed the use of NS only before athletes participated in competitions, our results might not accurately reflect their daily usage throughout the calendar year.

## Conclusion

About two-thirds of elite Japanese TF athletes reported the use of NS. Analyses by gender, age, and discipline showed that the prevalence of NS use was significantly higher in women, senior athletes, and long-distance runners than in others. The most prevalent items used by Japanese TF athletes were amino acids and vitamins, although the trends differed to some degree depending on the discipline. We also noted a gap between the prevalence of NS use and the evidence supporting their efficacy. Before consuming NS, athletes need to carefully examine the efficacy and safety of any products and consider their actual need.

## Data Availability

All the data from the current study is available on your request.

## Abbreviations

NS: Nutritional supplements
TF: Track and field
IOC: The International olympic committee
ISSN: The International society of sports nutrition
FDA: The US food and drug administration
ADRVs: Anti-doping rule violations
WADA: The World anti-doping agency
SD: Standard deviation
PMF: A pre-participation medical form
JAAF: The Japan association of athletics federations
JISS: The Japan institute of sports sciences
WA: World athletics
FIFA: The Fédération internationale de football association
BCAAs: Branched-chain amino acids
EAAs: Essential amino acids
JADA: The Japan anti-doping agency
